# Increased cardiovascular mortality in people with schizophrenia: a 24-year national register study

**DOI:** 10.1017/S2045796017000166

**Published:** 2017-06-05

**Authors:** J. Westman, S. V. Eriksson, M. Gissler, J. Hällgren, M. L. Prieto, W. V. Bobo, M. A. Frye, D. Erlinge, L. Alfredsson, U. Ösby

**Affiliations:** 1 Department of Neurobiology, Care Sciences, and Society, Division of Family Medicine, Karolinska Institutet, Stockholm, Sweden; 2 Department of Clinical Science, Danderyds Hospital, Stockholm, Sweden; 3 Department of Psychiatry and Psychology, Mayo Clinic, Rochester, MN, USA; 4 Facultad de Medicina, Departamento de Psiquiatría, Universidad de los Andes, Santiago, Chile; 5 Department of Cardiology, Lunds Universitet, Lund, Sweden; 6 Institute of Environmental Medicine, Karolinska Institutet, Stockholm, Sweden; 7 Center for Molecular Medicine, Karolinska Institutet, Stockholm, Sweden

**Keywords:** Epidemiology, psychosis, suicide, schizophrenia, myocardial infarction, mortality

## Abstract

**Aims:**

People who have schizophrenia die earlier from somatic diseases than do people in the general population, but information about cardiovascular deaths in people who have schizophrenia is limited. We analysed mortality in all age groups of people with schizophrenia by specific cardiovascular diseases (CVDs), focusing on five CVD diagnoses: coronary heart disease, acute myocardial infarction, cerebrovascular disease, heart failure and cardiac arrhythmias. We also compared hospital admissions for CVDs in people who had schizophrenia with hospital admissions for CVDs in the general population.

**Methods:**

This national register study of 10 631 817 people in Sweden included 46 911 people who were admitted to the hospital for schizophrenia between 1 January 1987 and 31 December 2010. Information from national registers was used to identify people who had schizophrenia and obtain data about mortality, causes of death, medical diagnoses and hospitalisations.

**Results:**

CVDs were the leading cause of death in people who had schizophrenia (5245 deaths), and CVDs caused more excess deaths than suicide. The mean age of CVD death was 10 years lower for people who had schizophrenia (70.5 years) than the general population (80.7 years). The mortality rate ratio (MRR) for CVDs in all people who had schizophrenia was 2.80 (95% confidence interval (CI) 2.73–2.88). In people aged 15–59 years who had schizophrenia, the MRR for CVDs was 6.16 (95% CI 5.79–6.54). In all people who had schizophrenia, the MRR for coronary heart disease was 2.83 (95% CI 2.73–2.94); acute myocardial infarction, 2.62 (95% CI 2.49–2.75); cerebrovascular disease, 2.4 (95% CI 2.25–2.55); heart failure, 3.25 (95% CI 2.94–3.6); and cardiac arrhythmias, 2.06 (95% CI 1.75–2.43). Hospital admissions for coronary heart disease were less frequent in people who had schizophrenia than in the general population (admission rate ratio, 0.88 (95% CI 0.83–0.94). In all age groups, survival after hospital admission for CVD was lower in people who had schizophrenia than in the general population.

**Conclusions:**

People who had schizophrenia died 10 years earlier from CVDs than did people in the general population. For all five CVD diagnoses, mortality risk was higher for those with schizophrenia than those in the general population. Survival after hospitalisation for CVDs in people who had schizophrenia was comparable with that of people in the general population who were several decades older.

## Background

People with schizophrenia have a 11 to 20 years shorter lifespan than people in the general population (Laursen *et al*. 2013). This decreased life expectancy may be caused by suicide or somatic illnesses, such as cardiovascular diseases (CVDs) (Brown *et al*. 2000; Osby *et al.* 2000; Nordentoft *et al.* 2013; Ösby *et al.* 2016).

People who have schizophrenia are more likely to have more than one major risk factor for CVDs; namely, overweight, smoking, high blood pressure and the metabolic syndrome (Deuschle *et al.* 2013; Gardner-Sood *et al.* 2015; Olsson *et al.* 2015; Gutiérrez-Rojas *et al.* 2016). However, they also are less likely to receive preventive care for these risk factors (Smith *et al.* 2013; Docherty *et al.* 2016). In addition, antipsychotic drugs, especially newer drugs, may cause major adverse cardiovascular events (Khasawneh & Shankar, 2014), but the character and magnitude of their exact effect on CVD risk are controversial (Tiihonen *et al.* 2009; Crump *et al.* 2013).

Although people who have psychotic disorders have twice the risk of CVD mortality as people in the general population (Crump *et al.* 2013; Nordentoft *et al.* 2013), little information is available about the age at which premature death occurs or the effects of specific CVDs. Such information would increase our understanding of the health care needs of people who have schizophrenia – needs that often are unmet.

National Health Service registers provide the opportunity to analyse specific mortality in all patients who had schizophrenia and were admitted to the hospital in Sweden from 1987 to 2010. We used data from National Health Service registers to compare CVD mortality in adults who had schizophrenia with that of adults in the general population by age and cause of death, focusing on five categories of CVDs: coronary heart disease, acute myocardial infarction, cerebrovascular disease, heart failure and cardiac arrhythmias. Additionally, we analysed mortality from other somatic causes, suicide and accidents. We placed particular focus on early deaths (deaths before age 60). We also compared hospital admissions for CVDs in people who had schizophrenia with hospital admissions for CVDs in the general population.

## Methods

### Cohort and follow-up

The study cohort included the 10 631 817 residents of Sweden during the 24 years between 1 January 1987 and 31 December 2010, including 46 911 people (0.44%) who were diagnosed with and admitted to the hospital for schizophrenia. To evaluate morbidity and mortality for each resident of Sweden during the study period, each person's unique personal identification number was used to link data from the Swedish Total Population Register, the Swedish National Patient Register (information about hospital admissions and medical diagnoses), and the Swedish National Cause of Death Register (information about date and causes of death).

The Swedish Total Population Register, established in 1968, contains information about the sex, date of birth, place of birth and date of migration of every resident in Sweden. The Swedish National Patient Register, maintained by the National Board of Health and Welfare, contains information about all hospital inpatient treatment in Sweden, including the unique personal identification number of each patient, dates of admission and discharge and diagnoses made during each hospitalisation.

All hospital diagnoses were classified in accordance with the World Health Organization's International Classification of Diseases (ICD). Diagnostic definitions changed substantially between the earlier 8th revision of the ICD (ICD-8) and the later revisions (ICD-9 and ICD-10). We therefore included only people diagnosed with schizophrenia in accordance with ICD-9 or ICD-10 criteria. Between 1987 and 1996, ICD-9 code 295 was used to identify schizophrenia diagnoses. From 1997 onward, ICD-10 codes F20 and F25 were used to identify schizophrenia diagnoses, including schizoaffective disorder.

The Swedish National Cause of Death Register includes data about all people who were registered in Sweden at the time of their death. The register provides information from death certificates about date of death and main (underlying) and additional causes of death. In the present study, we used only the main cause of death. Deaths due to CVD (ICD-9, 401–459; ICD-10, I10-I99) were subdivided into five groups of specific interest: deaths due to coronary heart disease (ICD-9, 410–414; ICD-10, I20-I25), including acute myocardial infarction (ICD-9, 410–411; ICD-10, I20), heart failure (ICD-9, 428; ICD-10, I50) and arrhythmia (ICD-9, 427; ICD-10, I45-I49) and deaths due to cerebrovascular disease (ICD-9, 430–438, ICD-10, I60-I69). Several subgroups with small numbers of people are not included in the tables and figures; therefore, the sum of included subgroup deaths is less than the total number of coronary heart disease deaths.

People with schizophrenia (*n* = 46 911) were followed up from the date of first hospital admission for schizophrenia until the earliest of three dates: date of death, date of emigration, or 31 December 2010. People in the general population (*n* = 10 631 817) were followed up from the date of their 15th birthday, immigration to Sweden, or 1 January 1987 (whichever came last) to the earliest of three dates: date of death, date of emigration or 31 December 2010.

### Statistical analysis

Data analysis was performed with statistical software (SAS, Version 9.4, SAS Institute Inc., Cary, NC, USA). Person-years, cause-specific deaths and hospital admissions caused by CVD were calculated and stratified by sex, calendar year and 5-year age groups. Mortality rate ratios (MRRs) and admission rate ratios (ARRs) were estimated with corresponding 95% confidence intervals (CIs) using Poisson regression models (Frome, 1983). The logarithm of the person-years of follow-up was used as the offset parameter. All models were adjusted for sex, age and calendar year of follow-up.

The expected number of deaths was determined by comparing the frequency of mortality in people in the general population with the frequency of mortality in people who had schizophrenia. Excess mortality in people who had schizophrenia was calculated as the difference between the observed and expected number of deaths.

When we calculated ARRs for CVD in people who had schizophrenia and people in the general population, follow-up ended on the day of first admission for CVD. For people with schizophrenia, as for people with CVD, mortality risk rises as time after diagnosis increases. We therefore used a 3-year washout period (1987–1989) to eliminate as many previous/old diagnoses of schizophrenia and CVD from our analyses as possible. That is, people who were admitted to the hospital for schizophrenia or CVD between 1987 and 1989 were excluded from the analyses.

## Results

### MRRs and causes of death

From 1987 to 2010, there were 13 895 deaths from all causes in people in Sweden who had schizophrenia (Table 1). There were 9462 excess deaths in people who had schizophrenia (reference group: the general population). An autopsy was performed to determine the cause of death in 5422 people who had schizophrenia (39%) and 422 113 people in the general population (19%) (data not shown).

**Table 1.** Mortality rate ratios and excess deaths in people who had schizophrenia in Sweden from 1987 to 2010*

	Men			Women			Total				
Cause of death	No. of deaths	MRR	95% CI	No. of deaths	MRR	95% CI	No. of deaths	MRR	95% CI	Excess deaths	95% CI
All causes of death	7259	3.36	(3.28–3.44)	6636	2.92	(2.85–2.99)	13 895	3.13	(3.08–3.19)	9462	(9231–9693)
Cardiovascular diseases	2731	3.09	(2.98–3.21)	2514	2.54	(2.44–2.64)	5245	2.80	(2.73–2.88)	3372	(3230–3514)
Coronary heart disease	1528	3.02	(2.87–3.18)	1143	2.61	(2.47–2.77)	2671	2.83	(2.73–2.94)	1728	(1627–1829)
Of which: Acute myocardial infarction	870	2.75	(2.57–2.94)	609	2.45	(2.26–2.65)	1479	2.62	(2.49–2.75)	914	(839–989)
Cerebrovascular disease	408	2.54	(2.31–2.80)	577	2.31	(2.13–2.50)	985	2.40	(2.25–2.55)	574	(513–636)
Heart failure	178	4.30	(3.71–4.98)	198	2.66	(2.32–3.06)	376	3.25	(2.94–3.60)	260	(222–298)
Cardiac arrhythmias	60	2.42	(1.87–3.11)	82	1.86	(1.50–2.31)	142	2.06	(1.75–2.43)	73	(50–96)
Other somatic diseases	3102	2.79	(2.70–2.89)	3347	2.78	(2.69–2.88)	6449	2.79	(2.72–2.86)	4136	(3979–4294)
Suicide	1047	11.75	(11.05–12.50)	552	18.12	(16.63–19.74)	1599	13.39	(12.73–14.08)	1480	(1401–1558)
Other external causes (injuries and poisoning)	379	4.15	(3.75–4.59)	223	4.05	(3.55–4.62)	602	4.11	(3.79–4.46)	456	(408–504)

* The reference group was the general population of Sweden.

CI, confidence interval; MRR, mortality rate ratio.

The most common cause of death in people who had schizophrenia was CVD; there were 3372 excess deaths from CVDs in people who had schizophrenia (reference group: people in the general population) (Table 1). Coronary heart disease (including acute myocardial infarction) and cerebrovascular disease were the two most frequent cardiovascular causes of death (Table 1). Acute myocardial infarction caused more than half the deaths from coronary heart disease (Table 1). The mean age of CVD death was 70.5 years for people who had schizophrenia and 80.7 years in the general population.

In people who had schizophrenia, there were 4136 excess deaths from other somatic diseases and 1480 excess deaths from suicide.

For people between the ages of 15 and 59 years, mortality was markedly higher in people who had schizophrenia than in the general population. This was true of mortality from all causes and from CVDs, including coronary heart disease, acute myocardial infarction and cerebrovascular disease (Table 2).

**Table 2.** Mortality rate ratios and excess deaths in people aged 15–59 years who had schizophrenia in Sweden from 1987 to 2010*

Cause of death	No. of deaths	MRR	95% CI	Excess deaths	95% CI
All causes of death	4415	5.74	(5.57–5.92)	3646	(3516–3776)
Cardiovascular diseases	1073	6.16	(5.79–6.54)	899	(835–963)
Coronary heart disease	596	6.11	(5.64–6.63)	499	(451–546)
Of which: Acute myocardial infarction	341	5.39	(4.84–6.00)	278	(242–314)
Cerebrovascular disease	97	3.07	(2.52–3.76)	65	(46–85)
Heart failure	27	13.56	(9.18–20.02)	25	(15–35)
Cardiac arrhythmias	22	8.23	(5.37–12.61)	19	(10–29)
Other somatic diseases	1660	3.80	(3.62–3.99)	1223	(1143–1303)
Suicide	1405	15.58	(14.76–16.45)	1315	(1241–1388)
Other external causes (unintentional injuries and poisoning)	277	4.13	(3.67–4.65)	210	(177–243)

* Men and women combined. The reference group was the general population of Sweden.

CI, confidence interval; MRR, mortality rate ratio.

**Fig. 1.**
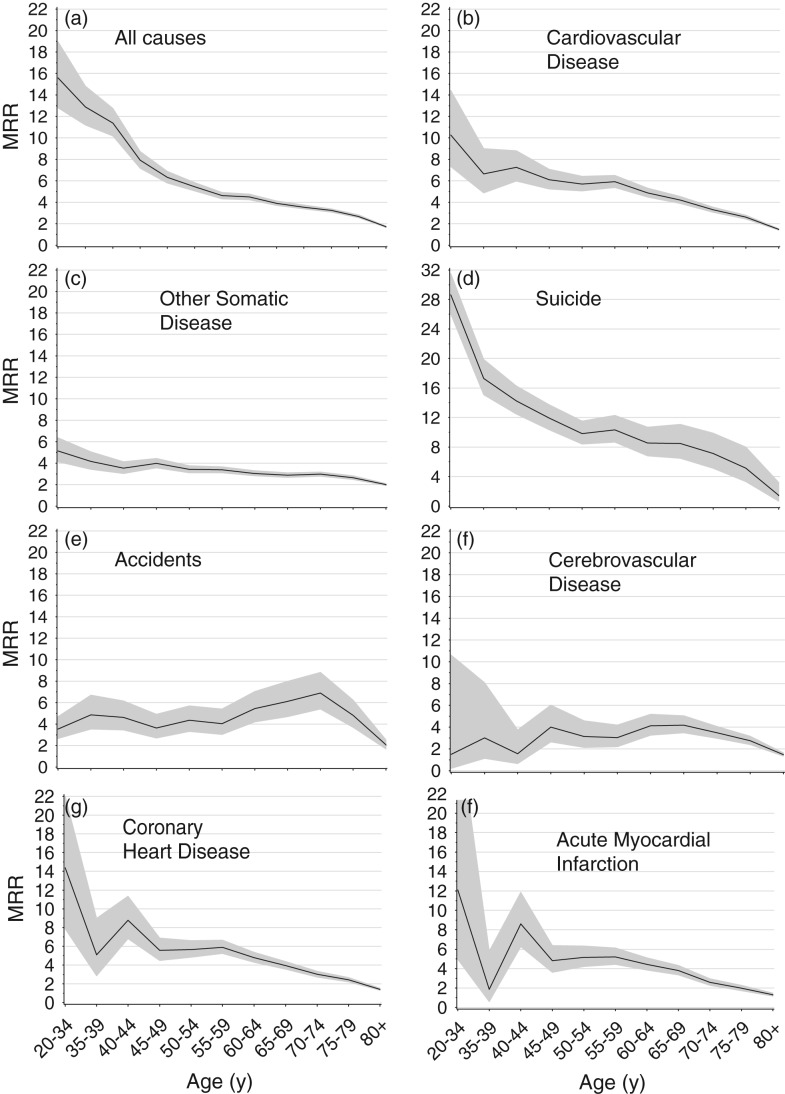
Mortality rate ratio (MRR) and age by cause of death in people who had schizophrenia in Sweden between 1990 and 2010. The reference group is the general population of Sweden. The black line represents the MRR, and the grey shading represents the 95% CI.

Mortality from CVDs occurred at a younger age in people who had schizophrenia than in the general population (Tables 3 and 4; Fig. 1).

**Table 3.** Relationship between age and cause of death in people who had schizophrenia in Sweden from 1987 to 2010*

	All causes of death			Cardiovascular diseases			Other somatic diseases			Suicide			Accidents		
Age (years)	No. of deaths	MRR	95% CI	No. of deaths	MRR	95% CI	No. of deaths	MRR	95% CI	No. of deaths	MRR	95% CI	No. of deaths	MRR	95% CI
15–19	15	21.29	(12.83–35.34)	0			1	4.67	(0.66–33.17)	13	77.77	(45.00–134.38)	1	3.38	(0.48–24.00)
20–24	101	15.58	(12.80–18.96)	1	3.37	(0.47–24.02)	5	2.87	(1.19–6.91)	91	41.61	(33.74–51.32)	4	1.64	(0.61–4.36)
25–29	201	12.88	(11.19–14.81)	8	7.64	(3.80–15.36)	20	3.78	(2.44–5.87)	162	30.52	(26.03–35.78)	11	2.50	(1.38–4.51)
30–34	316	11.38	(10.18–12.74)	25	10.19	(6.84–15.17)	60	5.30	(4.11–6.85)	198	22.91	(19.83–26.47)	33	5.62	(3.98–7.93)
35–39	399	7.92	(7.17–8.75)	42	6.63	(4.88–9.00)	101	4.15	(3.41–5.05)	218	17.30	(15.08–19.85)	38	4.86	(3.52–6.70)
40–44	551	6.32	(5.80–6.87)	107	7.25	(5.98–8.79)	168	3.55	(3.04–4.13)	229	14.25	(12.47–16.29)	47	4.61	(3.45–6.15)
45–49	741	5.43	(5.05–5.85)	176	6.09	(5.24–7.07)	320	3.98	(3.56–4.44)	202	11.91	(10.33–13.72)	43	3.64	(2.69–4.92)
50–54	908	4.61	(4.32–4.92)	273	5.70	(5.05–6.42)	420	3.42	(3.10–3.76)	162	9.83	(8.40–11.50)	53	4.34	(3.31–5.70)
55–59	1183	4.49	(4.24–4.75)	441	5.91	(5.37–6.49)	565	3.37	(3.10–3.66)	130	10.32	(8.66–12.30)	47	4.04	(3.03–5.38)
60–64	1307	3.89	(3.69–4.11)	532	4.88	(4.48–5.32)	640	3.04	(2.81–3.29)	77	8.54	(6.81–10.71)	58	5.43	(4.19–7.04)
65–69	1489	3.54	(3.37–3.73)	655	4.19	(3.88–4.52)	724	2.88	(2.68–3.10)	55	8.48	(6.49–11.08)	55	6.11	(4.68–7.98)
70–74	1722	3.23	(3.08–3.38)	738	3.29	(3.06–3.53)	883	2.97	(2.78–3.18)	36	7.12	(5.12–9.89)	65	6.89	(5.40–8.81)
75–79	1799	2.68	(2.56–2.81)	818	2.61	(2.43–2.79)	904	2.64	(2.47–2.82)	20	5.14	(3.31–7.98)	57	4.80	(3.70–6.23)
≥ 80	3163	1.71	(1.66–1.78)	1429	1.46	(1.39–1.54)	1638	2.00	(1.91–2.10)	6	1.43	(0.64–3.19)	90	2.07	(1.68–2.54)

* Men and women combined. The reference group was the general population of Sweden.

CI, confidence interval; MRR, mortality rate ratio.

**Table 4.** Relationship between age and specific cardiovascular causes of death in people who had schizophrenia in Sweden from 1987 to 2010*

	Cerebrovascular disease			Coronary heart disease			Acute myocardial infarction		
Age (years)	No. of deaths	MRR	95% CI	No. of deaths	MRR	95% CI	No. of deaths	MRR	95% CI
15–19	0	**–**	**–**	0	**–**	**–**	0	**–**	**–**
20–24	0	**–**	**–**	0	**–**	**–**	0	**–**	**–**
25–29	0	**–**	**–**	1	6.11	(0.85–43.94)	0	**–**	**–**
30–34	1	1.95	(0.27–13.92)	10	14.97	(7.94–28.22)	5	12.10	(4.95–29.55)
35–39	4	3.01	(1.12–8.07)	12	5.07	(2.86–8.98)	3	1.86	(0.60–5.79)
40–44	5	1.55	(0.64–3.74)	61	8.78	(6.80–11.34)	39	8.62	(6.26–11.86)
45–49	23	3.98	(2.64–6.02)	87	5.57	(4.50–6.89)	49	4.81	(3.63–6.38)
50–54	27	3.13	(2.14–4.57)	159	5.66	(4.83–6.62)	94	5.16	(4.21–6.33)
55–59	37	3.03	(2.19–4.19)	266	5.90	(5.22–6.66)	151	5.21	(4.43–6.12)
60–64	73	4.10	(3.26–5.17)	322	4.79	(4.29–5.35)	193	4.43	(3.85–5.11)
65–69	114	4.18	(3.47–5.03)	374	3.95	(3.56–4.37)	237	3.81	(3.36–4.33)
70–74	159	3.47	(2.97–4.06)	385	3.01	(2.72–3.32)	218	2.59	(2.27–2.96)
75–79	200	2.75	(2.39–3.16)	393	2.43	(2.20–2.68)	206	1.98	(1.72–2.27)
≥80	342	1.47	(1.32–1.64)	601	1.42	(1.31–1.54)	284	1.31	(1.17–1.47)

* Men and women combined. The reference group was the general population of Sweden.

CI, confidence interval; MRR, mortality rate ratio.

### Hospital admissions

In people with schizophrenia, there were more excess admissions for cerebrovascular disease than for either acute myocardial infarction or coronary heart disease (Table 5). People who had schizophrenia were not hospitalised for CVD at a frequency comparable to that of people in the general population (Table 5). Moreover, admissions to the hospital in people who had schizophrenia were not consistent with the high frequency of mortality from CVDs in this group.

**Table 5.** Hospital admissions in people who had schizophrenia in Sweden from 1990 t0 2010 after a 3-year washout period*

	Men			Women			Total			Total	
Cause of admission	*N*	ARR	95% CI	*N*	ARR	95% CI	*N*	ARR	95% CI	Excess admissions	95% CI
Coronary heart disease	476	0.87	(0.79–0.95)	374	0.90	(0.81–1.00)	850	0.88	(0.83–0.94)	−113	(−170 to −56)
Acute myocardial infarction	383	1.08	(0.98–1.20)	274	1.05	(0.93–1.18)	657	1.07	(0.99–1.15)	43	(−8 to 93)
Cerebrovascular disease	416	1.21	(1.10–1.33)	510	1.28	(1.17–1.40)	926	1.25	(1.17–1.33)	184	(124–243)

* The reference group was the general population of Sweden. We defined a 3-year washout period (1987–1989) to increase the number of people who had newly diagnosed schizophrenia and minimise potential confounding due to the high mortality associated with long-term hospitalisation

ARR, admission rate ratio; CI, confidence interval; N, number of admissions.

**Fig. 2.**
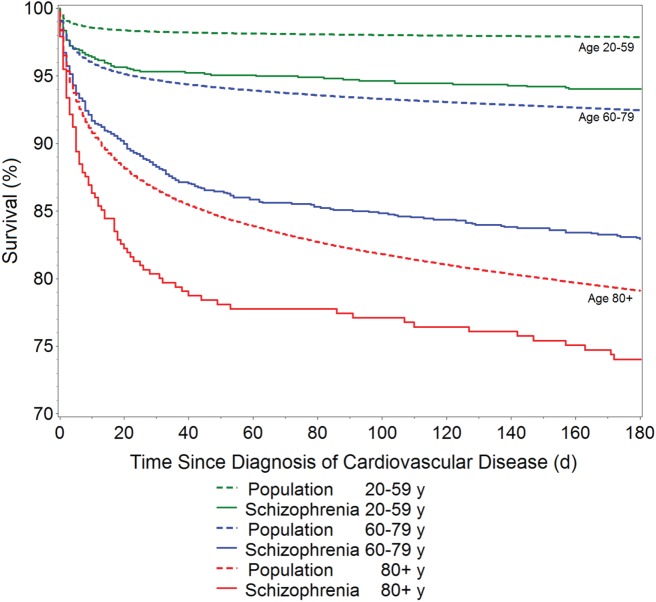
Survival after first hospital admission for cardiovascular disease in people who had schizophrenia and people in the general population in Sweden.

In all age groups, survival after first hospital admission for CVD was lower in people who had schizophrenia than in the general population (Fig. 2). Survival was similar for people aged 20–59 years who had schizophrenia and people aged 60–79 years in the general population, and survival was similar for people aged 60 to 79 years who had schizophrenia and people aged 80 years or older in the general population (Fig. 2).

## Discussion

This 24-year national register study showed that people who had schizophrenia had threefold higher mortality from CVDs than did people in the general population. For all five CVD diagnoses (coronary heart disease, acute myocardial infarction, cerebrovascular disease, heart failure and cardiac arrhythmias), mortality risk was higher for those with schizophrenia than those in the general population. CVD deaths typically occurred 10 years earlier in people who had schizophrenia. In people with schizophrenia, more excess deaths were caused by CVDs than by suicide. Frequency of hospital admission for CVDs was lower in people who had schizophrenia than in the general population.

This study used data about the entire population of Sweden aged 15 years or older. A major strength of the study was the use of register data with national coverage to evaluate the link between schizophrenia and premature cardiovascular mortality in a national cohort. The Swedish Cause of Death Register is of high quality because it covers more than 99% all deaths in Sweden. All causes of death included in the register are diagnosed by physicians in accordance with ICD-10. Cause-of-death codes on death certificates may be imprecise for various reasons (D'Amico *et al*. 1999; Chugh *et al.* 2004). However, in the present study, frequency of autopsy was twice as high in people who had schizophrenia than in the general population, which means that causes of death in people with schizophrenia were comparatively well-investigated.

A limitation of the present study was the exclusion of outpatient data, which was necessary to maintain uniform inclusion criteria throughout the study. The National Patient Register began to include psychiatric outpatient diagnoses in the mid-2000s. However, outpatient coverage in the register was too low during the early part of the study period to include these data in the study. Excluding outpatient data may have led us to miss people who had schizophrenia and had never been admitted to the hospital for the disorder. However, most people who have schizophrenia are treated at the psychiatric ward of a hospital soon after the onset of initial psychotic symptoms.

Another limitation of this study was that individual-level data about confounding factors such as smoking, alcohol consumption, exercise and other lifestyle factors could not be included in this study because they are not available in any of the Sweden's nationwide registers. Local primary care registers may include individual-level information about lifestyle, but it is typically incomplete. Lack of these data meant that we could not analyse the degree to which lifestyle factors explain the differences in CVD mortality between people with schizophrenia and people in the general population.

Previous studies have evaluated CVD-related deaths in people who had schizophrenia (Saha *et al.* 2007; Fan *et al.* 2013). A meta-analysis of 13 cohort studies showed that schizophrenia is significantly associated with an increased risk of mortality from CVDs (Fan *et al.* 2013). The present study adds new information about the age at which CVD death occurs in people who have schizophrenia and provides more detailed information about the magnitude of the problem. The present study showed that for people between the ages of 15 and 59 years, acute myocardial infarction was fivefold more frequent in those who had schizophrenia than in the general population. Furthermore, people who had schizophrenia were not hospitalised for their CVD at the same frequency as people in the general population. After hospital admission for CVD, those who had schizophrenia died more frequently than those in the general population, which suggests that CVD was inadequately treated in people who had schizophrenia.

As expected, we observed that people who had schizophrenia had increased suicide mortality. Although suicide frequency was 13-fold higher in people who had schizophrenia than in the general population, suicide caused fewer excess deaths (1480 excess deaths) than CVD (3372 excess deaths).

Although the data available in Swedish national registers did not enable us to adjust for lifestyle-related risk factors for CVD, other studies have shown that people who have schizophrenia are more likely to have at least one such risk factor, such as diabetes, smoking or hypertension (Gardner-Sood *et al.* 2015; Olsson *et al.* 2015). People who have schizophrenia are also less likely to receive good preventive somatic care than people without schizophrenia (Smith *et al.* 2013). In addition, antipsychotic drugs have been associated with adverse cardiovascular events (Mackin *et al.* 2008; Khasawneh & Shankar, 2014). Second-generation antipsychotic drugs may increase mortality via metabolic pathways that involve weight gain, diabetes and dyslipidaemia (Newcomer & Haupt, 2006; Ray *et al.* 2009). These are known problems and therefore important to take into account in monitoring and treatment (Buckley *et al.* 2005; Barnes *et al.* 2007; Morrato *et al.* 2008; Mitchell *et al.* 2012). However, some studies have shown no association between clozapine or other commonly used antipsychotic drugs and increased mortality (Crump *et al.* 2013) or a negative association (Tiihonen *et al.* 2009).

In summary, this large national Swedish register study showed that those with schizophrenia had higher mortality from CVDs than those in the general population. Additionally, the study provides new and more detailed information about this mortality. First, mortality risk from five CVD diagnoses was higher in people with schizophrenia than in people in the general population. Second, the risk of dying from CVDs was six times higher in younger people (under 60 years) with schizophrenia; deaths from CVDs occurred 10 years earlier in people with schizophrenia than in people in the general population. Third, CVDs were the main reason for premature death in people who had schizophrenia, accounting for more excess deaths than suicide. Fourth, in people with schizophrenia, survival after hospitalisation for CVDs was similar to that of people in the general population who were decades older. The large number of excess deaths, low hospital admission frequency and poor survival after first hospital admission indicate that CVDs were undertreated in people who had schizophrenia, especially younger people.

## Availability of data and materials

All register information was provided by the Swedish National Board of Health and Welfare. Information about how to apply for data from Swedish health care registers is available at www.socialstyrelsen.se.
